# BioModels linked dataset

**DOI:** 10.1186/s12918-014-0091-5

**Published:** 2014-08-15

**Authors:** Sarala M Wimalaratne, Pierre Grenon, Henning Hermjakob, Nicolas Le Novère, Camille Laibe

**Affiliations:** European Molecular Biology Laboratory, European Bioinformatics Institute (EMBL-EBI), Wellcome Trust Genome Campus, Hinxton, Cambridge, CB10 1SD UK; CHIME, The Farr Institute of Health Informatics Research, London, NW1 2DA UK; Babraham Institute, Babraham Research Campus, Cambridge, CB22 3AT UK

**Keywords:** BioModels database, Semantic web, Resource description framework, Linked data, SPARQL

## Abstract

**Background:**

BioModels Database is a reference repository of mathematical models used in biology. Models are stored as SBML files on a file system and metadata is provided in a relational database. Models can be retrieved through a web interface and programmatically via web services. In addition to those more traditional ways to access information, Linked Data using Semantic Web technologies (such as the Resource Description Framework, RDF), is becoming an increasingly popular means to describe and expose biological relevant data.

**Results:**

We present the BioModels Linked Dataset, which exposes the models’ content as a dereferencable interlinked dataset. BioModels Linked Dataset makes use of the wealth of annotations available within a large number of manually curated models to link and integrate data and models from other resources.

**Conclusions:**

The BioModels Linked Dataset provides users with a dataset interoperable with other semantic web resources. It supports powerful search queries, some of which were not previously available to users and allow integration of data from multiple resources. This provides a distributed platform to find similar models for comparison, processing and enrichment.

## Background

The number of mathematical models of biological processes published over the last decade has grown in part due to standard format and software tool development efforts in the Systems Biology community. BioModels Database [1] was developed to support the storage, search and retrieval of these models. It provides around 1200 models published in the scientific literature (a large portion of which are manually curated) and over 142,000 models automatically generated from pathway resources [2] such as from KEGG [3], BioCarta [4], MetaCyc [5] SABIO-RK [6] and PID [7].

The core format used by BioModels Database is the Systems Biology Markup Language (SBML) [8], a widely used computer-readable format for representing computational models in biology. In an effort to unambiguously identify model elements and understand their relation with the biological processes they describe, they are extensively annotated with cross-references to external resources such as Gene Ontology [9], ChEBI [10], Reactome [11] and UniProt [12]. The relationship between each annotated model component and the accompanying cross-reference to an external resource is specified using terms from a controlled vocabulary [13].

The model files are stored in a file system, while metadata, such as elements’ name and annotations, are stored in a SQL database. This enables convenient querying of the repository’s content and retrieval of models of interest. The database also supports programmatic access to its content through web services.

Making data available as Linked Data using the Resource Description Framework (RDF) [14] is becoming a popular method for integrating resources [15]. RDF is based on a simple triple concept, subject-predicate-object, which allows users to capture detailed or abstract concepts. An RDF-based data model is essentially a collection of RDF statements. The statements form a linked structure where two labeled nodes (the subject and the object) is linked via a named edge (the predicate).

RDF triples are often hosted in a triple store, a purpose-built database for the storage and retrieval of triples. For example, OpenLink Virtuoso [16] is an open source triple store for storing large RDF graphs. APIs are available for extracting data and generating RDF graphs, such as Apache Jena [17], an open source Semantic Web framework for Java which provides an API for handling RDF graphs. SPARQL [18], a query language for databases that are able to retrieve and manipulate data stored in RDF format, also supports complex queries that merge data distributed over multiple RDF resources hosted at different physical locations.

To enable wider access and better integration with data across multiple repositories, we have exposed the models from BioModels Database in RDF. This effort, named the BioModels Linked Dataset is part of an EBI wide effort to support semantic integration of bioinformatics data [19]. This effort includes other EMBL-EBI resources such as UniProt, ChEMBL [20], Gene Expression Atlas [21], Reactome, and BioSamples [22].

## Method

### SBML to RDF conversion

The definition of a SBML model consists of lists of one or more components. This includes *Compartment*, *Species*, *Reaction*, *Parameter*, *Unit definition*, and *Rule*. These are represented in XML using *ListOfX* elements (for example *ListOfSpecies*) which are used to list each of the corresponding elements found in the entirety of the model. XML attributes are used to represents parameters such as *name*, *identifier*, etc.

The conversion of an SBML model into RDF is performed by representing each SBML element (*Compartment*, *Species*, *Reaction*, *Parameter*, *Unit definition,* etc.) with a corresponding class from the BioModels RDF Vocabulary. Each of these is represented as a subclass of the *SBMLElement* class, which itself is a subclass of *Element*. The generic *Element* class was introduced to extend this structure to other formats, such as CellML [23].

The SBML XML attributes are captured using RDF properties. For example attributes such as *name* (associated with most SBML elements), *initialAmount* and *initialConcentration* (associated with the *Species* element) are captured as properties with the same names.

The *ListOfX* element, which is used to group concepts in SBML, is not represented in RDF. Instead the content of these elements is directly linked to the parent resource using RDF properties, and RDF typing is used to type each class. This simplifies the RDF representation and improves query performance.

Similarly, the *Annotation* element, which is used to add RDF content to any SBML element, is not represented, but its content is provided in a simplified way, by removing the *rdf:bag* tags. This allows the resulting RDF to provide direct relationship between a SBML element (e.g. a Species) and its annotations using BioModels qualifiers.

An additional attribute, *curated*, was introduced to capture whether a particular model had undergone the resource’s manual curation process (ensuring model reproducibility and further reuse).

Since current use cases focus on the linking of model component data, rather than the simulation behavior of the model itself, the mathematical constructs present within the SBML models has been omitted. Also, converting mathematical constructs into OWL/RDF is a complex exercise, a research topic that needs to be addressed separately. This means that the RDF representation of the models should not be seen as a replacement for SBML representation, but more a complement to be used in specific cases. The BioModels Linked Dataset enables users to find and explore relevant models using Semantic Web technologies easily, while the SBML encoded models are used for exchange and simulation purposes.

The complete BioModels RDF Vocabulary used here is available at: http://identifiers.org/biomodels.vocabulary, and a diagrammatic representation of the schema is available at: http://www.ebi.ac.uk/rdf/documentation/biomodels. Table 1 lists the prefixes and the namespaces that are used in this paper to denote different statements.

**Table 1:** **Prefixes and namespaces used in this paper**

**Prefix**	**Namespace**
**biodb**	http://identifiers.org/biomodels.db/
	BioModels Database namespace
**sbmlrdf**	http://identifiers.org/biomodels.vocabulary
	BioModels Linked Dataset vocabulary
**bqbiol**	http://biomodels.net/biology-qualifiers
	BioModels.net biology qualifiers
**bqmodel**	http://biomodels.net/model-qualifiers
	BioModels.net model qualifiers
**cco**	http://rdf.ebi.ac.uk/terms/chembl#
	CHEMBL Core Ontology
**atlasterms**	http://rdf.ebi.ac.uk/terms/atlas/
	Atlas Linked Dataset vocabulary

A detailed explanation of the BioModels Linked Dataset structure using specific examples is provided below. It describes how *Species* and *Reactions* elements are captured in RDF and illustrates the resulting triples.

**Figure 1:**
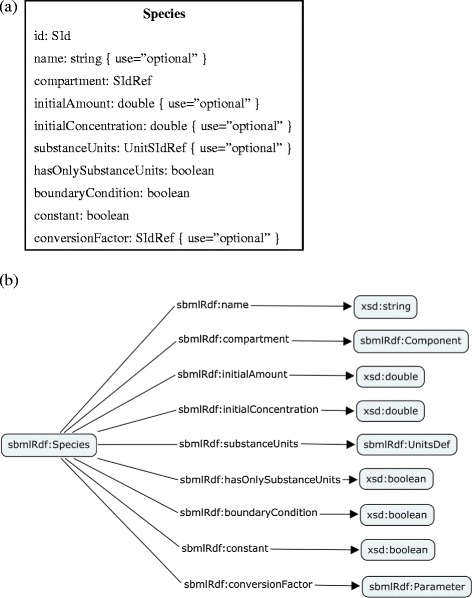
**SBML species definition. (a)** Diagrammatic representation of the SBML XML Species class definition. **(b)** Diagrammatic representation of the SBML RDF Species definition. Classes are illustrated using boxes and the arrow denotes the property relationship between the classes.

An SBML Species represents a pool of entities (Figure 1a). Such entities are considered indistinguishable from each other for the purposes of the model, may participate in reactions, and are located in a specific compartment. The SBML *Species* element is represented as a class *Species* in RDF. Its attributes, for instance the XML attribute *name,* are captured as properties in RDF (Figure 1b).

The RDF triples generated through such a species conversion are illustrated below, using http://identifiers.org/biomodels.db/BIOMD0000000001#_000003 as an example. All RDF snippets are expressed using the TURTLE format.

**Figure 2:**
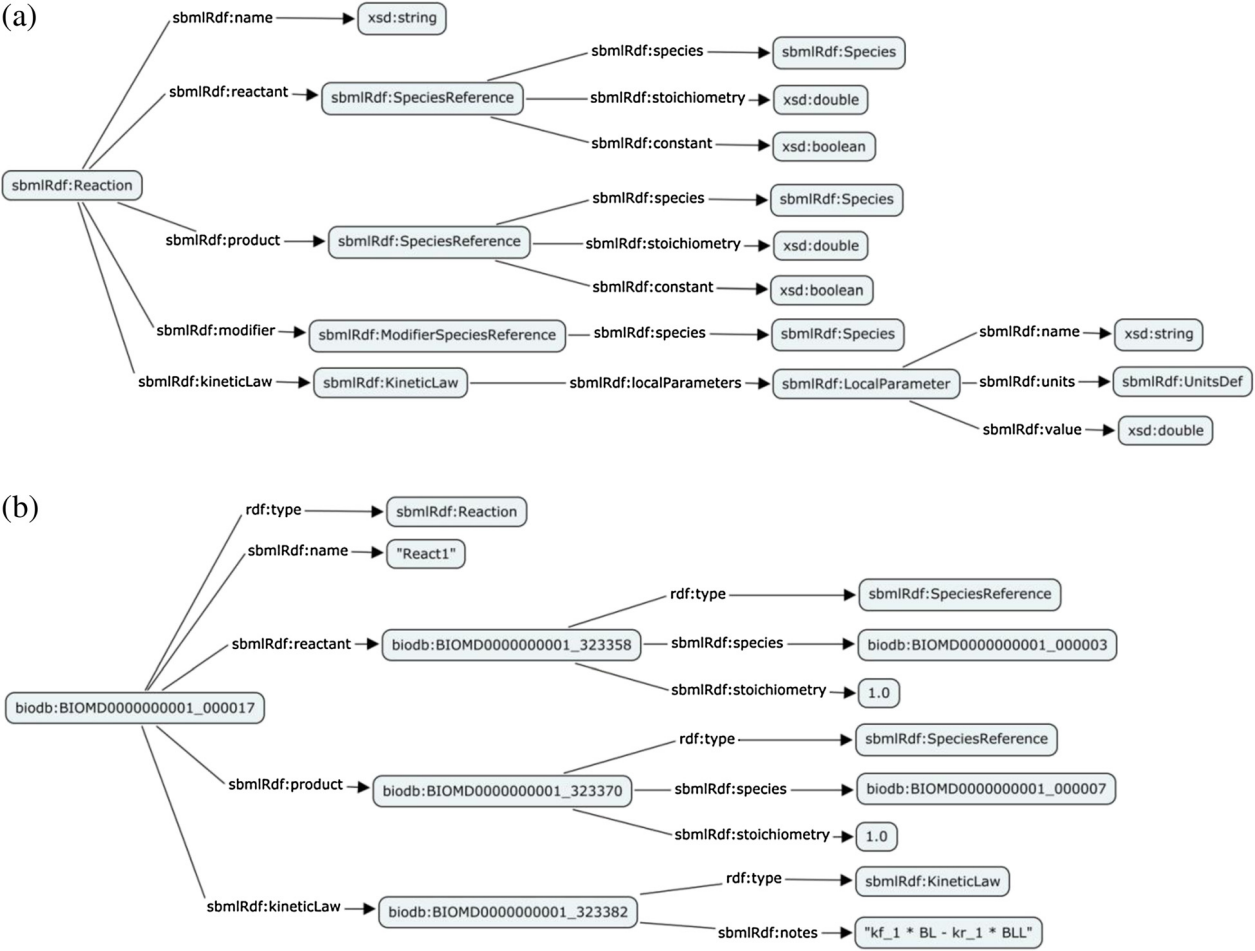
**SBML reaction definition. (a)** Diagrammatic representation of the SBML RDF Reaction definition. Classes are illustrated using boxes and the arrow denotes the property relationship between the classes. **(b)** Diagrammatic representation of instantiation of a reaction, http://identifiers.org/biomodels.db/BIOMD0000000001#_000017.

An SBML Reaction represents any kind of process that can change the quantity of one or more species in a model. Figure 2a illustrates the RDF schema that was created for the Reaction. Figure 2b shows an instantiation of the Reaction class to capture the following reaction: BasalACh↔BasalACh2.

Each species participating in a reaction as a substrate or product is declared using a SpeciesReference. The SpeciesReference class has the property sbmlrdf:species to reference the participating species. Currently mathematical equations are captured as strings using the property sbmlrdf:notes. Following this conversion, the triples generated for the example reaction above are:

### URIs

Each RDF model is identified using a unique resolvable Uniform Resource Identifier (URI) provided by Identifiers.org [24]. Specific model elements are uniquely identified using a leading hash combined with the SBML meta id; for example http://identifiers.org/biomodels.db/BIOMD0000000008#202866. In addition to providing a globally unique way to identify models, Identifiers.org URIs also enable direct access to models by just resolving the URL (Figure 1).

References to external resources are made using Identifiers.org URIs, and in cases where canonical URIs are available, owl:sameAs statements are added to record them too. This provides a greater degree of interoperability with other linked datasets.

### Storage and provision

To enable semantic query of such data, it is necessary to populate a triple store with the RDF statements resulting from the previously described conversion. We use OpenLink Virtuoso for storing BioModels Linked Dataset. Query access to the dataset is provided by exposing the triple store through a SPARQL endpoint that can be accessed at: http://www.ebi.ac.uk/rdf/services/biomodels/sparql.

### Distribution

RDF files are regenerated with each BioModels Database release (2 to 4 times a year), and distributed with the other model archives, and the Virtuoso triple store is repopulated with the new files. This means that the triple store only contains the latest release and does not keep track of the old revisions.

### System architecture

The system is running on two independent data centers. Each datacenter consists of instances of a Virtuoso repository and a LODEStar application [25]. The LODEStar application provides a simple interface for querying and browsing the RDF triples.

## Results

This paper describes the BioModels Linked Dataset using BioModels Database release 27, from Apr 2014. The SBML and RDF files for this release can be downloaded from: ftp://ftp.ebi.ac.uk/pub/databases/biomodels/releases/2014-04-11/. This includes all models published in the literature, together with the SBML RDF schema and a dataset description which contains metadata about the dataset. The source code for the SBML to RDF converter can be found at https://github.com/sarala/ricordo-rdfconverter. A set of example SPARQL queries are detailed at http://www.ebi.ac.uk/rdf/documentation/biomodels/queries, which includes all the queries described in this paper.

### Data statistics

The BioModels Linked Dataset consists of an RDF representation of all models from BioModels Database’s literature branches (excluding the Path2Models branches). It includes 529 curated and 655 non-curated models, comprising 18,960,824 triples, and 1,805,055 cross-references pointing to 82,157 different biological concepts.

The dataset includes 441,008 species in total. This number can be obtained via the following SPARQL query:

It is also possible to calculate the distinct number of species using the annotations. The following query returns 55,080 species (note: this does not include species without annotations).

The dataset includes 885,004 reactions representing 23,671 distinct reactions deduced using the annotations. Queries can also be performed to collect statistical information, such as the number of model elements annotated to a given resource.

### Example local queries

Simple queries can be written to list individual elements such as Species or Reactions within a particular model. For example, the query below will list all the species (with their names) which appear in the model *Edelstein1996 - EPSP ACh event* (BIOMD0000000001):

Queries can be constructed to identify elements that relate to specific concepts using ontological annotations. The example below finds all model elements linked to the gene ontology term, *acetylcholine-gated channel complex* (GO:0005892):

SPARQL also allows pattern matching. For example, it is possible to write a query to find all model elements that have annotations to any Reactome pathways. The following query does just that and returns the model elements, element type, qualifier and the specific Reactome annotation:

It is possible to run more complex queries using SPARQL. For example query for models which describe reactions involving calcium ions in the cytosol of rat. The following query looks up models that have annotations to rat (http://identifiers.org/taxonomy/10114), cytosol (http://identifiers.org/goo/GO:0005829), and calcium ion (http://identifiers.org/chebi/CHEBI:29108). The results list models and their reactions that have elements with annotations to all three concepts.

### Federated queries

**Figure 3:**
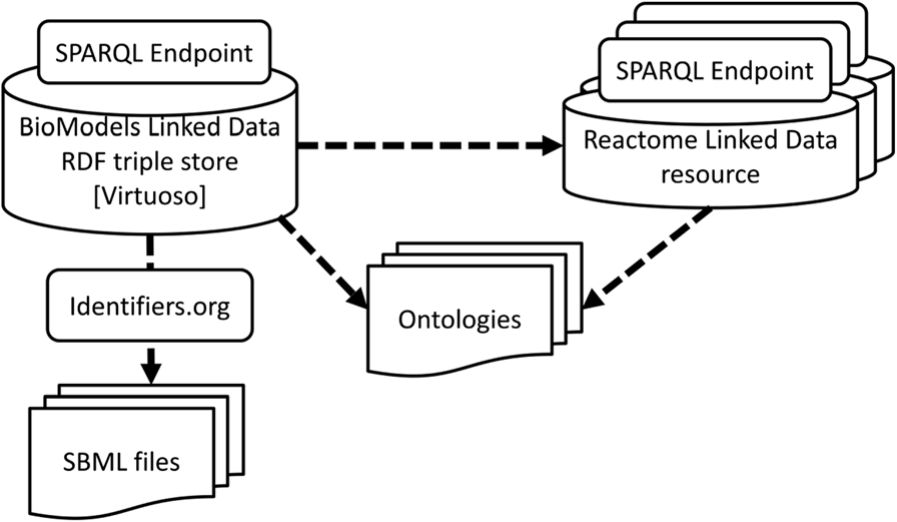
**BioModels linked dataset mapping.** Dashed lines illustrate the direct links that exist within the dataset to other data resources and ontologies. This enables queries that span across multiple data stores via the SPARQL Endpoints. Access to individual models can be achieved via Identifiers.org URIs.



Most bioinformatics resources cross reference each other and have direct references to ontological terms (Figure 3). Using these mappings, it is possible to construct a query which retrieves and integrates information from different resources. For example one can query for ChEMBL protein targets present in a particular model. This can be achieved by writing one query which integrates data from BioModels Database and ChEMBL via the common cross references to UniProt proteins. The query below first looks up model elements with annotations to UniProt. The result is then used to query ChEMBL Linked Dataset via its SPARQL endpoint to retrieve ChEMBL targets:

Using external links, one can query models using resources that are not directly used to annotate models. For instance both BioModels Database and Expression Atlas [26] provides direct extensive annotations to UniProt proteins, but provides no cross-references to each other. Using these common UniProt cross references, it is possible to run a query to find all model elements that relate to a particular gene, *Tgfbr2* (ENSMUSG00000032440, transforming growth factor, beta receptor II) even if BioModels Database does not have models directly annotated with *Tgfbr2*. This query first looks up in the Atlas Linked Dataset to find relevant UniProt identifiers. The result is then used to query BioModels Linked Dataset to find relevant model elements:

BioModels Linked Dataset can be used to compare models. For example: finding models to compare with annotations to some proteins that are used as drug targets by lactic acid molecule. The query below returns two models that link to proteins which can be used as target proteins for the lactic acid molecule. Without BioModels Linked Dataset, one would have to write a small program which would call many different web services to achieve this result. Note that model comparison will be done manually, while finding models to compare with is done via one SPARQL query.

## Discussion

BioModels Linked Dataset provides a unique interface to query both the content and semantics of models. This solution also allows execution of federated queries across other RDF repositories, providing a powerful mechanism to integrate heterogeneous data.

The tools provided so far by BioModels Database, such as the web search, and SOAP based web services, could not answer some of the queries described in this paper. For example, queries across elements from multiple models would previously require a user to download all the models locally and run some custom scripts to extract the necessary information.

BioModels web services [27] provide several methods that can be used to retrieve all models annotated with commonly used resources such as GO, UniProt, Taxonomy, ChEBI and Reactome. For example, *getSimpleModelsByReactomeIds* method retrieves all the models which are annotated with the given Reactome records. However, it is not possible to retrieve all models that have any Reactome annotation. As described in the results section BioModels Linked Dataset could be used to execute such queries.

In addition to a richer data query and access method, BioModels Linked Dataset provides users with an interoperable dataset, which can be accessed using standard Semantic Web technologies, such as SPARQL. Previous efforts have explored semantic data integration of SBML models [28,29]. They mainly focused on transforming the SBML files into biological models encoded in the Web Ontology Language [30]. These representations were generated from the model annotations and enabled complex ontological reasoning over the resulting dataset.

Our approach focuses on linking and integrating data across multiple data sources to extract more information about the models. It relies on some concepts from the RICORDO framework [31], which illustrates querying BioModels Linked Dataset through intermediate reasoning over ontologies used for annotating resources [32]. This allows users to run complex queries, such as ‘find all models which have annotations to some part of membrane’.

Future plans include the integration of the Path2Models [2] branches of BioModels Database into the BioModels Linked Dataset.

## Conclusions

Exposing BioModels Database content through Semantic Technologies makes the knowledge captured within individual annotations more widely accessible and discoverable. This information can be used to link data and models seamlessly across multiple resources, thereby facilitating complex query across multiple such resources, through a simple interface. Hence, this provides a novel and useful addition to the current set of services provided by BioModels Database.

Moreover, being developed as part of the EBI RDF effort, the BioModels Linked Dataset is built upon a stable and powerful infrastructure for the storage and query of RDF triples.

Ultimately, this new offering from BioModels Database enables the semantic web community to cross query between EBI and others datasets as one large web of data.

### Ethical approval

This work does not require ethical approval.
